# Mitochondrial dynamics regulate cell morphology in the developing cochlea

**DOI:** 10.1242/dev.202845

**Published:** 2024-08-09

**Authors:** James D. B. O'Sullivan, Stephen Terry, Claire A. Scott, Anwen Bullen, Daniel J. Jagger, Zoë F. Mann

**Affiliations:** ^1^ Centre for Craniofacial and Regenerative Biology, King's College London, 27th Floor, Guy's Tower, London SE1 9RT, UK; ^2^ UCL Ear Institute, University College London, 332 Gray's Inn Rd, London WC1X 8EE, UK; ^3^ The London Centre for Nanotechnology, University College London, 17-19 Gordon Street, London WC1H 0AH, UK

**Keywords:** Metabolism, Mitofusin, Biogenesis, Hearing, Chick

## Abstract

In multicellular tissues, the size and shape of cells are intricately linked with their physiological functions. In the vertebrate auditory organ, the neurosensory epithelium develops as a mosaic of sensory hair cells (HCs), and their glial-like supporting cells, which have distinct morphologies and functional properties at different frequency positions along its tonotopic long axis. In the chick cochlea, the basilar papilla (BP), proximal (high-frequency) HCs, are larger than their distal (low-frequency) counterparts, a morphological feature essential for sound perception. Mitochondrial dynamics, which constitute the equilibrium between fusion and fission, regulate differentiation and functional refinement across a variety of cell types. We investigate this as a potential mechanism for regulating the shape of developing HCs. Using live imaging in intact BP explants, we identify distinct remodelling of mitochondrial networks in proximal compared with distal HCs. Manipulating mitochondrial dynamics in developing HCs alters their normal morphology along the proximal-distal (tonotopic) axis. Inhibition of the mitochondrial fusion machinery decreased proximal HC surface area, whereas promotion of fusion increased the distal HC surface area. We identify mitochondrial dynamics as a key regulator of HC morphology in developing inner ear epithelia.

## Teaser

**Highlighted Article:** Mitochondrial remodelling drives developmental changes in cell morphology in the auditory sensory epithelium. This study reveals a fundamental mechanism regulating cell morphology and frequency-place coding in the developing cochlea.

## INTRODUCTION

**Fig. 1.**
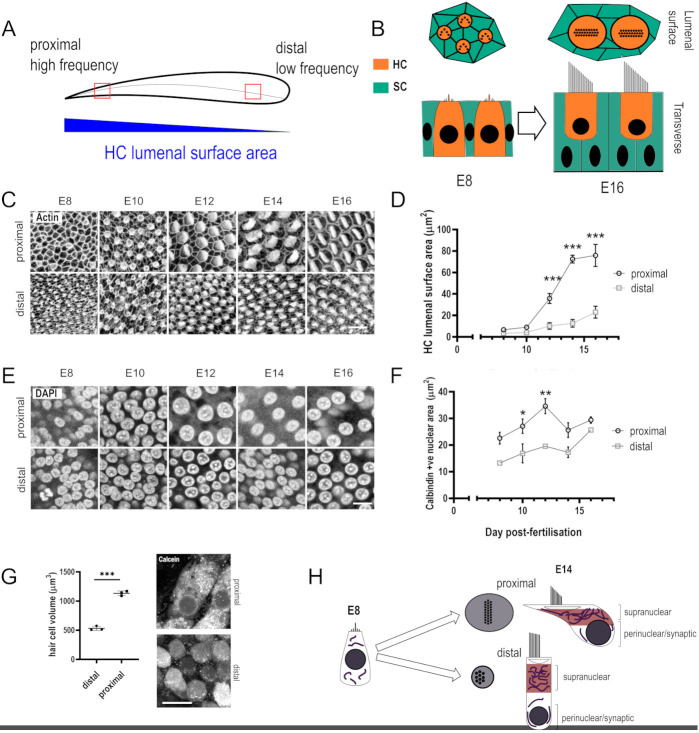
**Tonotopic variations in hair cell morphology along the developing chick cochlea.** (A) Schematic of the avian auditory sensory epithelium. Hair cells (HCs) are arranged longitudinally along the organ according to frequency sensitivity, and this arrangement (tonotopy) is correlated with a gradient in HC lumenal surface area. The location of imaging ROIs used for all proximal-to-distal comparisons are indicated by red squares. (B) Changes in the structure of the auditory sensory epithelium between E8 and E16. HCs are surrounded by a mosaic of glial-like supporting cells (SCs). Top: surface view showing the lumenal surface area at the apical pole of the HC. Bottom: transverse cross-section of HCs. (C) Whole mount Phalloidin staining of proximal and distal BP HCs between E8 and E16. (D) Quantification of HC luminal surface area in proximal and distal regions between E8 and E16. Data are mean±s.d., *n*=3 embryos, 15-40 HCs per embryo. ***P≤0.001, two-way ANOVA, Sidak's multiple comparisons, *F*=1354, DF=3. (E) Whole mount DAPI staining of proximal and distal HCs between E8 and E16. (F) Quantification of HC nuclear area between E8 and E16. Data are mean±s.d., *n*=3 embryos, *n*=15-40 HCs per embryo. *P>0.05, **P>0.01, two-way ANOVA, Sidak's multiple comparisons, *F*=45.74, DF=3. (G) Live imaging of calcein-AM loaded E14 explants from which HC volume was estimated using stereology. Data are mean±s.d., *n*=3. ***P<0.001, unpaired two-tailed *t*-test, *t*=19.03, DF=4. (H) Changes in HC morphology between E8 and E14 in proximal and distal BP regions. The supranuclear region of HCs (red) that, in proximal HCs, undergoes a burst of growth between E10 and E14 in the supranuclear relative to the perinuclear region. The supranuclear region contains a high density of mitochondria. Scale bars: 20 µm (C); 10 μm (E,G).

A unique feature of the avian cochlear epithelium is that the position of a hair cell (HC) along its proximal-distal long axis determines the sound frequency to which it will respond, a phenomenon known as ‘tonotopy’ (Mann and Kelley, 2011). The tonotopic patterning of the avian cochlea (the basilar papilla; BP) is characterised in part by a proximal-to-distal gradient in HC morphology (Tilney and Saunders, 1983) (Fig. 1A). High-frequency HCs at the proximal end of the BP have a higher surface area at their apical pole (the endolymph-facing lumenal surface area; LSA) than the low-frequency distal HCs at the opposite end. Specification of an HC's ‘tonotopic identity’ relies on the coordinated signalling between graded glucose metabolism and morphogen signalling before their terminal differentiation at around embryonic day (E) 6-E8 (Mann et al., 2014; Thiede et al., 2014; Son et al., 2015; O'Sullivan et al., 2023). Once instructed of their tonotopic identity, little is known about the effector mechanisms that generate the morphological differences between proximal and distal HCs in the mature epithelium.

Mitochondrial dynamics, referring to the distribution of mitochondria throughout a cell and remodelling of their networks by fusion and fission, have emerged as a key determinant of cell size and function (Baum and Gama, 2021; Fu et al., 2019). The dynamic equilibrium between fusion and fission also determines the morphological and metabolic properties of individual mitochondria. Biases of the mitochondrial network towards fission or fusion are unique to different cell types and tailored to support their specific functions. Fusion and fission are coordinated by a set of mitochondrial proteins (Van Der Bliek et al., 2013). The mitofusins 1 and 2 (MFN1 and MFN2) regulate dynamics of the outer mitochondrial membranes (Chen et al., 2003), Optic Atrophy 1 (OPA1) coordinates crista structure and inner mitochondrial membrane fusion (Patten et al., 2014; Wong et al., 2000), and Dynamin 1-like (DNM1L; Drp1 in mammals) separates individual mitochondria through the formation of a contractile ring (Chang et al., 2010; Ji et al., 2017). Asymmetric fission regulates mitochondrial quality control by segregating compromised mitochondria from the rest of the network and targeting them for removal via mitophagy (Song et al., 2015). Fusion promotes mitochondrial longevity and lessens the energetic load imposed on a single organelle (Fu et al., 2019).

Mounting evidence implicates mitochondrial dynamics as a regulator of the signalling pathways driving cell fate decisions during development (Khacho et al., 2016). For example, fission regulates differentiation in neural progenitors and morphological remodelling of T-cells during activation (Cervantes-Silva et al., 2021; Iwata et al., 2020). Increased mitochondrial fusion is also important for specifying the distinct morphologies of neurons and astrocytes during neural development (Zehnder et al., 2021). Increased mitochondrial biogenesis and fusion has long been considered a consequence of the higher metabolic demands of larger cells (Kitami et al., 2012; Posakony et al., 1977), a hypothesis supported by work showing that biasing certain cell lines towards fusion increases their overall volume (Miettinen and Björklund, 2016). The physical size of cells alters their functionality, making cell-size-dependent metabolism relevant for understanding development, disease and aging in tissues.

Here, we use the tonotopic differences in HC LSA (Fig. 1A) along the chick BP as a model in which to interrogate the metabolic mechanisms regulating the gradient in cell morphology established during development. We find that mitochondrial dynamics and network architecture are extensively remodelled in BP HCs, and that biasing towards fusion or fission within a crucial window of development alters the HC morphology associated with frequency position, an important aspect of tonotopy.

## RESULTS AND DISCUSSION

To understand how HC morphology changes during development, the HC LSA was characterised between E8, when the majority of HCs are terminally differentiated, and at E16, when HCs are morphologically and functionally mature (Lippe, 1995) in proximal and distal regions of the BP (Fig. 1A,B). We observed no tonotopic difference in LSA at E8 or E10, but differential growth was evident along the tonotopic axis between E10 and E14 (Fig. 1C,D). These differences in growth plateaued between E14 and E16, identifying E14 as a potential growth endpoint in developing HCs. In addition to measuring HC LSA, we used DAPI and calbindin staining to further probe graded differences in HC size along the BP. DAPI and calbindin staining revealed that, in contrast to LSA, proximal HCs had larger nuclei than distal HCs as early as E10, although this tonotopic difference was lost by E14 (Fig. 1E,F). Tonotopic differences in HC volume were estimated using 3D imaging of calcein-loaded HCs in live BP explants (Fig. 1G). Contrary to previous studies in HCs from the mammalian cochlea (Fettiplace and Nam, 2019), analysis of calcein fluorescence revealed that, by E14 (growth endpoint), the volume of proximal HCs was roughly twice that of distal HCs (Fig. 1G). The high ratio of LSA growth compared with nuclear growth and overall cell volume suggest that the cuticular plate region/LSA towards the apical pole of the HC (hereafter referred to as supranuclear region) exhibits accelerated growth compared with the rest of the cell during development (Fig. 1H).

HCs contain a variety of spatially distinct mitochondrial sub-populations (Fig. S1A), the majority residing in the supranuclear region towards the apical portion of the cell. Given the asymmetric shape of HCs and their unique morphology along the tonotopic axis, we sought to investigate how the mitochondrial dynamics in the HC supranuclear region differ along the proximal-to-distal axis between E8 and E14. Transmission electron microscopy (TEM) in proximal and distal HCs revealed that mitochondria became progressively thicker and acquired denser laminar cristae arrangements between E8 and E14 (Fig. S1B), indicating a higher capacity for oxidative phosphorylation with developmental progression (Cogliati et al., 2013). To determine whether mitochondrial dynamics correlate with the tonotopic gradient in supranuclear growth, we investigated potential differences in the remodelling of mitochondrial networks in developing HCs along the proximal-to-distal axis.

**Fig. 2.**
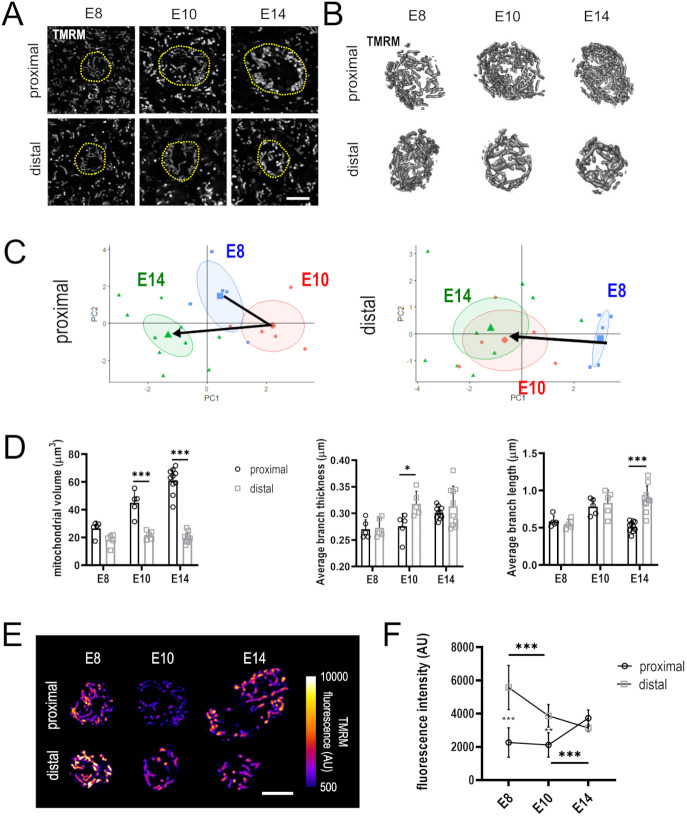
**Mitochondrial morphology and membrane potential in developing HCs.** (A) Example *z*-slices from explants at E8, E10 and E14 after loading with the mitochondrial dye TMRM. *Z*-stacks of the supranuclear mitochondrial population were acquired using TMRM fluorescence. HCs are delineated by yellow dashed lines. (B) 3D models of supranuclear mitochondrial networks within HCs. Images were processed for noise reduction and edge enhancement. (C) Individual PCA plots of proximal and distal HC mitochondrial morphology, as observed from live (Airyscan) microscopy of TMRM-loaded BP explants dissected at E8, E10 and E14. Mitochondrial morphology in proximal HCs differs significantly between E8, E10 and E14 (shaded circles show 95% confidence intervals), compared with distal HCs in which mitochondrial morphology at E10 and E14 is comparable. Individual measurements which explain the variables contributing to PCs 1 and 2 are detailed in D. (D) Quantification of individually measured mitochondrial shape descriptors. Mitochondrial volume was not included in the PCA. Data are mean±s.d., *n*=6, 6, 11 HCs per timepoint. *P≤0.05, ***P≤0.001, two-way ANOVA followed by Sidak's multiple comparisons. (E) Airyscan images of mitochondria from proximal and distal HCs in explants loaded with 350 nM TMRM at E8, E10 and E14. Images are single confocal slices of HC mitochondria demonstrating changes in HC mitochondrial membrane potential throughout development (E8-E14). (F) At E8 and E10, mitochondria in distal HCs have a significantly higher membrane potential than those in proximal HCs. By E14, mitochondrial membrane potentials have equilibrated and become similar. Data displayed as mean±s.d., *n*=6 HCs from four independent biological replicates. **P≤0.01, ***P≤0.001, two-way ANOVA, Sidak's multiple comparisons, *F*=46.85, DF=18. Scale bars: 5 µm (A,E).

To visualise changes in supranuclear mitochondrial morphology throughout development, live cochlear explants were loaded with the fluorescent mitochondrial dye tetramethyl rhodamine methyl ester (TMRM) between E8 and E14 (Fig. 2), encompassing all stages of HC maturation (Si et al., 2003). Raw fluorescence intensity data were processed and segmented to generate 3D representations of supranuclear mitochondrial networks (Fig. S3) from which average branch thickness, standard deviation of branch thickness, maximum branch thickness, average branch length, maximum branch length, longest shortest path, Euclidean distance (a measure of branch tortuosity) and branch aspect ratio were calculated. Principal Component Analysis (PCA) (Wiemerslage and Lee, 2016) of these eight measurements revealed that HC mitochondria developed along distinct morphological trajectories in proximal and distal regions (Fig. 2A-D; Fig. S1C). Mitochondrial morphology in both regions was comparable at E8, indicated by the high overlap of PCA clusters (Fig. S1C). Mitochondrial volume in proximal HCs increased between E8 and E14 (Fig. 2D), suggesting greater mitochondrial fusion and overall network growth (Miettinen and Björklund, 2016) in the high-frequency compared with low-frequency region. At E10, proximal and distal mitochondria segregated largely based on differences in branch thickness (Fig. 2D; Fig. S1C). Thicker mitochondrial branches typically accommodate more lamellar cristae, which increases their capacity for ATP production (Afzal et al., 2021). Given the distal-to-proximal gradient in cell cycle exit during development (Goodyear and Richardson, 1997) the thicker mitochondrial branches in distal HCs at E10 could reflect a more differentiated metabolic state (Spurlock et al., 2020). At E14, the average mitochondrial branch length was shorter in proximal compared with distal HCs (Fig. 2D). We attribute this to the higher overall network density and close proximity of multiple adjacent mitochondria in high frequency HCs. Owing to this, individual mitochondria could not be accurately separated. The further remodelling of mitochondrial dynamics in proximal compared with distal HCs between E10 and E14 indicates more extensive metabolic reprogramming in high frequency HCs as they develop. This is likely a consequence of the distinct physiological properties emerging between the two HC types at this time (Fettiplace and Nam, 2019).

To investigate how mitochondrial activity, mitochondrial content and HC cell LSA scale at different tonotopic positions, we quantified the fluorescence intensity of TMRM-loaded mitochondria, itself a read-out of the mitochondrial membrane potential (ΔΨm) and thus mitochondrial activity (Duchen et al., 2003). Unexpectedly, we observed that, despite a similar mitochondrial morphology at E8, distal HC mitochondria displayed higher TMRM fluorescence and thus ΔΨm compared with proximal HCs, which was maintained until E10 (Fig. 2E,F). Although increased branch length is typically associated with increased mitochondrial activity, our observations suggest that in developing HCs, mitochondrial morphology and membrane potential are not tightly coupled. Furthermore, by E14, when proximal and distal mitochondria have largely different morphologies, ΔΨm had equalised between the two HC types (Fig. 2B). These findings suggest that, at least in HCs, ΔΨm and mitochondrial activity are not isometrically scaled with their dynamics and morphology. The lower ΔΨm observed in proximal HCs at E8 and E10 may result from reduced activity in the mitochondrial tricarboxylic acid (TCA) cycle, occurring because of increased glucose flux into the pentose phosphate pathway (PPP) in proximal HCs at this time. Higher glucose flux into the PPP would reduce mitochondrial pyruvate uptake, slow metabolic flux into the TCA cycle and, as a consequence, lower ΔΨm (O'Sullivan et al., 2023). Another hypothesis could be that by uncoupling mitochondrial content from activity as seen in other cell types (Miettinen et al., 2017), developing HCs can simultaneously double the mitochondrial content and control their growth rate during the cell cycle.

**Fig. 3.**
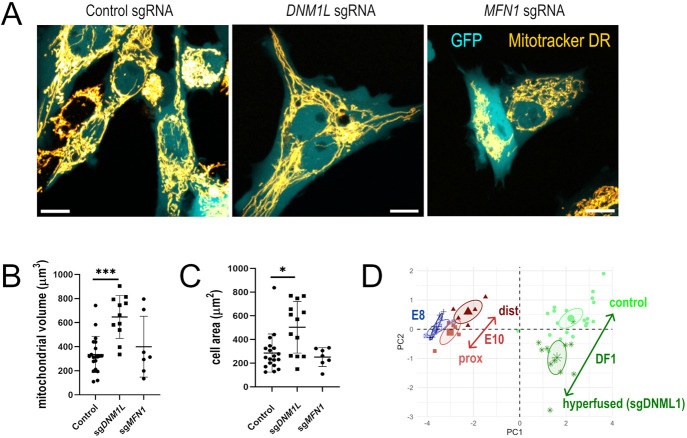
**CRISPR-Cas9 knockout of fusion and fission regulators in DF-1 cells.** (A) Maximum intensity projections of confocal *z*-stacks of GFP (cytosol) and MitoTracker DR (mitochondria) in DF-1 cells. (B) Total mitochondrial volume was increased in cells transfected with sgDNM1L compared with controls. No difference was observed in sgMFN1-transfected cells. Data are mean±s.d. *P<0.05, ***P<0.001, Kruskal–Wallis, Sidak's multiple comparisons; statistic=14.03, DF=26. (C) Cell area was increased in DF-1 cells transfected with sgDNM1L compared with controls. No difference was observed in sgMFN1 cells. Data are mean±s.d. *P<0.05, Kruskal–Wallis, Sidak's multiple comparisons; statistic=8.742, DF=26. (D) Morphometric analysis of control and sgDNM1L cell mitochondria reveals that their characteristics differ along the same axis as E10 proximal and distal HCs. Scale bars: 5 µm.

Previous work has shown that biasing cells towards fusion increases their volume (Miettinen and Björklund, 2016). We directly tested this hypothesis using a chick fibroblast cell-line. Chicken DF-1 fibroblasts were transfected with plasmids containing a customisable CRISPR-Tol2 construct expressing an sgRNA guide and GFP reporter and Cas9 (Daudet et al., 2022) that allowed the expression of the mitochondrial regulators *MFN1* and *DNM1L* to be manipulated (Fig. 3A). Following knockout (KO) of *DNM1L*, which should inhibit fission and promote fusion, fibroblasts exhibited higher mitochondrial volume (Fig. 3B) and an overall larger cell area (Fig. 3C) compared with control fibroblasts, confirming that a bias towards fusion favours a larger cell size. When the morphological characteristics of mitochondria from *DNM1L* KO and control fibroblasts were compared with the PCA analysis conducted for E8 and E10 HCs, the difference in morphological trajectory between *DNM1L* KO and control fibroblasts matched those observed between proximal and distal HCs at E10 (Fig. 3D). This supports the hypothesis that biasing mitochondrial dynamics towards fusion in proximal HCs could drive the tonotopic differences in supranuclear growth and HC size.

**Fig. 4.**
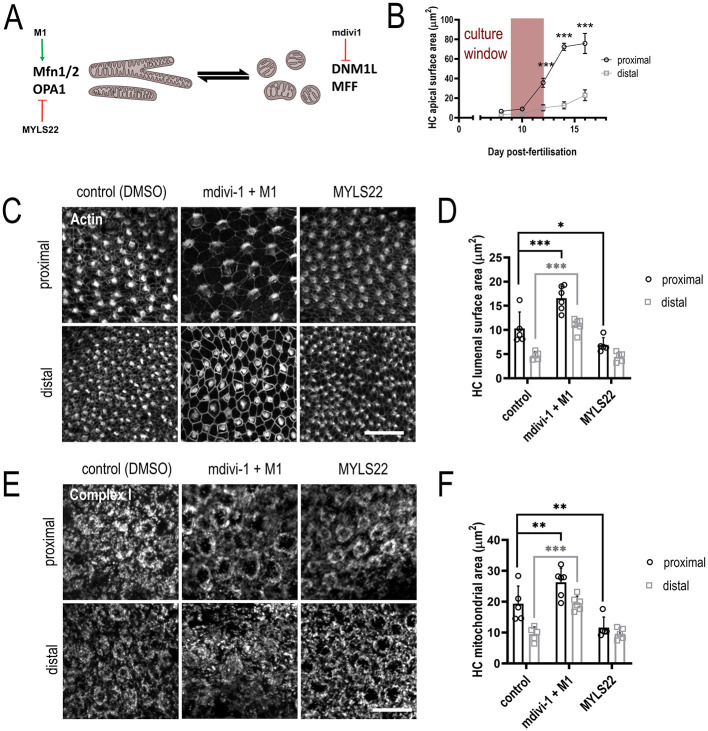
**Functional interrogation of mitochondrial dynamics in the developing chick cochlea.** (A-F) Cochleae were dissected from embryonic chickens at E9. The sensory epithelium was isolated and cultured *ex vivo* for 4 days with small molecule modulators of mitochondrial dynamics. HC apical surface area was delineated using Phalloidin staining, and the area of mitochondrial immunoreactivity with anti-Complex I antibody was measured above the apex of the nucleus. (A) Illustration depicting the site of action of different small molecule modulators. Mdivi-1 inhibits DNM1L and therefore fission. M1 promotes fusion. MYLS22 inhibits inner mitochondrial membrane fusion via OPA1. (B) Quantification of *in vivo* changes of HC size (E8-E16) reproduced from Fig. 1D. The time window for explant culture experiments is indicated by the pink band. (C) Effects of mitochondrial dynamics modulators on HC apical surface area. M1 and mdivi1 in combination results in a substantial increase in distal HC surface area. Conversely, treatment with MYLS22 reduced apical HC surface area. (D) Quantification of modulator effects shown in C, *n*=5, 6, 5 independent biological replicates. Data are mean±s.d. mdivi1+M1: ***P<0.001, two-way ANOVA, Sidak's multiple comparisons, *t*=5.242, DF=26. MYLS22: *P<0.05, two-way ANOVA, Sidak's multiple comparisons, *t*=2.698, DF=26. (E) Effects of mitochondrial dynamics modulators on HC mitochondrial area in the supranuclear region. Mitochondrial area was quantified in the slice above the apical tip of the nucleus. M1 and mdivi1 in combination results in an increase in distal mitochondrial area. Conversely, treatment with MYLS22 reduced proximal mitochondrial area. (F) Quantification of modulator effects shown in E, *n*=5, 6, 5 independent biological replicates. Data are mean±s.d. mdivi1+M1: ***P<0.001, **P<0.01, two-way ANOVA, Sidak's multiple comparisons, *t*=4.529, DF=26. MYLS22: **P<0.01, two-way ANOVA, Sidak's multiple comparisons, *t*=3.356, DF=26. Scale bars: 20 µm (C); 10 µm (E).

We next sought to functionally examine a role for mitochondrial dynamics in regulating HC morphology at the extreme ends of the tonotopic axis using pharmacological inhibitors to manipulate fusion and fission (Fig. 4A) during the period of postmitotic cell growth between E9 and E12 (Fig. 4B). Treatment with the fission inhibitor mdivi-1 (Cassidy-Stone et al., 2008) or the fusion promoter M1 (Wang et al., 2012) alone did not induce notable changes in the HC LSA (Fig. S2A,B). However, co-treatment with mdivi-1 and M1 increased the distal HC LSA to levels comparable with those of proximal HCs (Fig. 4C,D). Inhibition of fusion with MYLS22, an inhibitor of OPA1 (Baek et al., 2023; Herkenne et al., 2020), reduced HC LSA in the proximal BP region to levels comparable with distal HCs. Moreover, the effects of M1+mdivi-1 and MYLS22 on LSA were closely correlated with changes in the area of mitochondrial complex 1 staining in the supranuclear region of each measured HC, indicating that the effects on LSA were positively associated with changes in mitochondrial abundance (Fig. 4E,F). Taken together, these data suggest that blocking fusion is sufficient to prevent supranuclear growth in proximal HCs and that a proximal-to-distal gradient in mitochondrial fusion and mitochondrial volume may establish the tonotopic gradient in HC LSA.

Overall, our findings suggest that differences in mitochondrial fusion in the supranuclear region of HCs are a driver, rather than a consequence, of the larger high-frequency HC LSA. These morphological differences manifest as distinct surface-volume (SV) ratios at different tonotopic positions. Despite a lack of direct experimental evidence showing a causative link between the HC SV ratio and its physiological membrane properties in birds, there is good evidence that, in mammals, tonotopic position correlates with HC size. In mammals, HC length also correlates inversely with ion channel density such that shorter HCs have higher potassium channel numbers per unit membrane area (Johnson et al., 2011; Mammano et al., 1995). We therefore suggest that in the chick cochlea, mitochondrial regulation of the HC SV ratio may play an important role in determining their physiological properties and electrical tuning during development (Fettiplace and Nam, 2019; Fuchs et al., 1988; Johnson et al., 2011). To confirm a functional role for mitochondrial dynamics on tonotopic development in HCs it will be necessary to examine whether manipulating fusion and fission impacts stereociliary bundle morphology, the kinetics and expression profile of potassium channels or the transducer current along the tonotopic axis. Another interesting question is whether intracellular signalling pathways associated with cellular growth such as mTOR (Lloyd, 2013; Morita et al., 2017) regulate mitochondrial morphology differently between basal and apical regions of the HC to determine their asymmetric shapes.

Our work has identified mitochondrial fusion as a regulator of HC size, highlighting another key function of mitochondria in the ear. Furthermore, we demonstrate how insights from primary cell lines (Miettinen and Björklund, 2016) and our own experiments can be interpreted to understand cell size regulation in complex tissues.

## MATERIALS AND METHODS

### Experimental animals

Fertilised chicken eggs (Dekalb white breed) were obtained from a commercial supplier (Medeggs, Fakenham, UK) and incubated at 37°C before experiments. All procedures were performed in accordance with the United Kingdom Animals (Scientific Procedures) Act (1986).

### CRISPR-Cas9 knockout constructs

pCAGG-NLS-Cas9-NLS (expressing nuclear localised *Streptococcus pyogenes* Cas9 under the expression of CAGC promoter) was a gift from Marianne Bronner (Addgene plasmid #99138; Gandhi et al., 2017).

Tol2-U6.3-sgRNA-empty-GFP (Addgene plasmid #221843) (expressing within a Tol2 transposon a cytoplasmic GFP reporter under the control of GAGC promoter and sgRNA guide expressed under chick-specific U6.3 promoter) was made by first amplifying EGFP from PCMS28 EGFP IRES Puro (Terry et al., 2018) via PCR using primers containing 5′ EcoRI (gaattcGCCACCATGGTGAGCAAGG) and 3′ BamHI (ggatccTTACTTGTACAGCTCGTCCATG) sites (underlined sections show the restriction enzyme sequences for EcoRI and BamHI added to the primer sequences). Tol2-U6.3-sgRNA-tdTomato (made by VectorBuilder) was cut using EcoRI-BamHI to excise tdTomato and the EcoRI-EGFP-BamH1 PCR product was ligated into this vector. sgRNA guide sequences were designed using https://genome-euro.ucsc.edu/ with the March 2018 GRCg6a/galGal6 *Gallus gallus* genome release and chosen with the following parameters: MIT specificity score>80%, efficiency score>80% (Doench et al., 2016) and out-of-frame score>65 (Bae et al., 2014). sgRNA were made by annealing pairs of synthesised complementary oligos (Eurofins-MWG) containing 20 base pair sgRNA guide sequences with additional four nucleotide overhangs (GGAT on forward, AAAC on reverse) for directional cloning into Tol2-U6.3-sgRNA-GFP at BsaI sites. sgRNA sequences are as follows: for non-targeting control (GCACTGCTACGATCTACACC) Tol2-U6.3-sgRNA-non-targeting-control-GFP (Addgene plasmid #221844), for *DNM1L* (gTGTTTTCCGACCATCCTCTG) targeting exon 2 Tol2-U6.3-sgRNA-DNM1L-GFP (Addgene plasmid #221845) and for *MFN1* (GAGAAGAAGAGCGTCAAGGT) targeting exon 3, Tol2-U6.3-sgRNA-MFN1-GFP (Addgene plasmid #221846).

### Transfection of DF-1 cells with CRISPR-Cas9 plasmid constructs

DF-1 fibroblasts were cultured in DMEM high glucose, GlutaMAX with sodium pyruvate (Thermo Fisher Scientific, 10569010) supplemented with 10% fetal bovine serum at 5% CO_2_ 37°C. DF-1 cells (50,000 per well) were plated into 24-microwell imaging plates (ibidi, 82426). Cells were transfected 24 h later using lipofectamine 3000 (Thermo Fisher Scientific, L3000008) with 1 μg of plasmid NLS-Cas9-NLS and 0.5 μg of appropriate Tol2-sgRNA-GFP plasmids. Cells were imaged live 72 h after transfection. We purchased DF-1 cells directly from ATCC (chicken DF-1 fibroblast cells, CRL-12203, ATCC) and screened routinely for any evidence of mycoplasma.

### Explant culture

The auditory sensory epithelium was dissected from chicken embryos at E9. Explants were adhered to glass-bottomed Mattek dishes using 20% v/v CellTak solution according to the manufacturer's instructions. Tissue explants were cultured for 96 h in M199 medium supplemented with 2% bovine serum albumin and 10 mM HEPES at 37°C in 5% CO_2_. For small molecule treatments, stock solutions of MYLS22 (Cambridge Bioscience, HY-136446), mdivi-1 (Merck, M0199) and M1 (Merck, SML0629) in DMSO were diluted in medium to their final concentrations. Medium for control treatments was supplemented with 0.2% DMSO (vehicle). Following the treatment protocol, explants were fixed for 20 min in 4% paraformaldehyde (PFA) in PBS (pH 7.2).

### Histochemistry and immunohistochemistry

Tissue was harvested from embryos for immunohistochemistry of calbindin, and explant cultures were prepared for immunohistochemistry of complex 1. The otic capsule was dissected from E8, E10 and E14 embryos and then fixed in 4% PFA in PBS (pH 7.2) for 1 h, before dissection of the auditory sensory epithelium. Both freshly dissected tissue and explant cultures were then permeabilised with 0.3% Triton X-100. Blocking was performed with 10% goat serum in 0.3% Tween 20, before overnight incubation with unconjugated primary antibodies at 4°C: Calbindin (Abcam, Monoclonal Ms ab82812-1001, 1:75 and lot no. 1027333-1, 1:75); Complex 1 (Invitrogen, Monoclonal Ms 18G12BC2, lot no. YH4011265, 1:100). Tissue was stained with an Alexa Fluor 488-conjugated secondary antibody at room temperature for 1 h (Thermo Fisher Scientific, A11001, 1:1000), before post-staining with DAPI and Phalloidin-633 (Thermo Fisher Scientific, A22284) to detect DNA and F-actin, respectively. Finally, samples were mounted on eight-well slides with Prolong-gold mountant (Thermo Fisher Scientific, P36930).

For measurements of LSA, a region of interest (ROI) of 50 µm×50 µm was assigned to the centre of each image. The apical actin ring of each HC within the ROI was then manually traced, before an average of all HC areas was calculated for each sample. For measurements of mitochondrial area made from immunofluorescence of complex 1, a single optical slice positioned just above the nucleus was selected, within the same ROI used for LSA measurements. The top 65% of the fluorescence histogram was then thresholded for each image, and the mitochondrial area for each HC was measured.

### Transmission electron microscopy

Cochleae were dissected and fixed in 2.5% glutaraldehyde in 0.1 M cacodylate buffer (pH 7.4) and were then bisected into proximal and distal portions before post-fixation in 1% buffered osmium tetroxide. Following post-fixation, samples were dehydrated to 70% ethanol before *en bloc* staining with 1% uranyl acetate in 70% ethanol. Samples were further dehydrated in ethanol before substitution with 100% propylene oxide. The samples were then infiltrated with increasing concentration of TAAB 812 hard resin, and mounted in the resin, which was cured according to the manufacturer's instructions.

Then, 70 nm ultra-thin sections were taken using a Reichert Ultracut ultramicrotome. Ultra-thin sections were then stained with 1% aqueous uranyl acetate and 1% lead citrate before imaging at 8000× magnification in a Jeol 1400 electron microscope with a 120 kV beam energy.

### Live imaging

Auditory sensory epithelia were dissected at E8, E10 and E14 and loaded with 350 nM TMRM (Thermo Fisher Scientific, T668) in L-15 medium for 1 h at room temperature. E14 tissue was dissected and loaded in a 1000× diluted stock solution of CellTrace Calcein red-orange AM as indicated by the manufacturer (Thermo Fisher Scientific, C34851). Dye-loaded explants were imaged in Mattek dishes on a Zeiss 980 confocal microscope, using the 561 nm laser and Airyscan 2 detector. *Z*-stacks were acquired, with an optical slice thickness of 18 nm. Airyscan processing was performed to software-recommended settings. DF-Fibroblasts were imaged 72 h after transfection and loaded with 100 nM MitoTracker Deep Red (MTDR; Thermo Fisher Scientific, M22426) for 30 min at 37°C in DMEM. Cells were imaged on a Zeiss 880 confocal microscope using a 488 nm laser for GFP and 633 nm laser and Airyscan 2 detector for MTDR.

### Cell volume

To aid throughput, cell volume was estimated using a stereology approach similar to that described by Spring and Hope (1979). For each HC, a cross-section was annotated manually every 0.9 µm slice interval of the *z*-stack (one in every five slices starting from the top of the cell and moving progressively down), and its area was measured. For each HC analysed, the output was therefore a register of cross-sectional areas taken at regular intervals through the *z*-stack. As the optical section was 0.18 μm, each cross-sectional area was multiplied by 5 to obtain an estimate of the volume of each segment of the HC in the register. For each HC, the volume segments were added together to generate an estimate of the total volume of each cell.

### Mitochondrial network morphology

Raw data files were processed in the Fiji distribution of ImageJ (Schindelin et al., 2012) (Fig. S3). To ensure compatibility with the ImageJ skeleton module, images were first resampled to achieve isotropic pixel size using the CLIJ2 3D resample operation (Haase et al., 2020). Subsequently, background fluorescence was eliminated using ‘subtract background’ using a rolling ball size of 50 pixels. An unsharp masking filter was then applied, followed by the application of a non-local means filter. Next, a difference of Gaussian filter was applied using minimum and maximum sigma of 2 and 4 pixels, respectively. Overall, we found this processing pipeline productive for minimising background and enhancing mitochondrial edges, leading to greater consistency between segmentation of different datasets.

Mitochondria were labelled using 3D WEKA segmentation. First, we carried out manual segmentation of data on a single confocal slice and used this to train a classifier. The classifier was then used to segment mitochondria from the rest of the data analysed.

Binary stacks were generated from the 3D WEKA segmentation output. Next, individual HCs were cropped from the binary image stacks using the ImageJ ROI tool, such that a single binary stack was generated for each HC in the dataset. Skeletons were generated for each HC image using the Skeleton extension. The BoneJ plugin (Doube et al., 2010) was used to analyse the binary stack from each HC, while the Analyse Skeleton plugin was used to analyse skeleton stacks to output branching information. From these two outputs, seven measurements of local mitochondrial network characteristics were made which were subsequently visualised by PCA. These eight measurements consisted of: average branch thickness, standard deviation of branch thickness, maximum branch thickness, average branch length, maximum branch length, longest shortest path, Euclidean distance (a measure of branch tortuosity) and branch aspect ratio. These local mitochondrial characteristics were measured in individual HCs and their developmental trajectories were visualised in the PCA and compared between proximal and distal regions. Mitochondrial networks were plotted along principal components 1 and 2, providing graphical representation of these developmental trajectories.

### Statistical analysis

PCA was carried out in R and visualised using the factoextra package. For statistical analysis of live imaging, histochemical and immunohistochemistry experiments, data were tested for normality and Q-Q plots were visually inspected to determine application of parametric and non-parametric tests. A mixture of two-way ANOVA, unpaired two-tailed *t*-tests and Kruskal–Wallis tests with appropriate post-hoc tests were carried out in Prism 8 (GraphPad Software).

## Supplementary Material



10.1242/develop.202845_sup1 Supplementary information
