# Supplementary material for: Mitochondrial dynamics regulate cell morphology in the developing cochlea
Source: Development. 2024 Aug 9;151(15):dev202845. doi: 10.1242/dev.202845 (PMC11809207; doi:10.1242/dev.202845)
Supplement: Supplementary information [file develop-151-202845-s1.pdf]

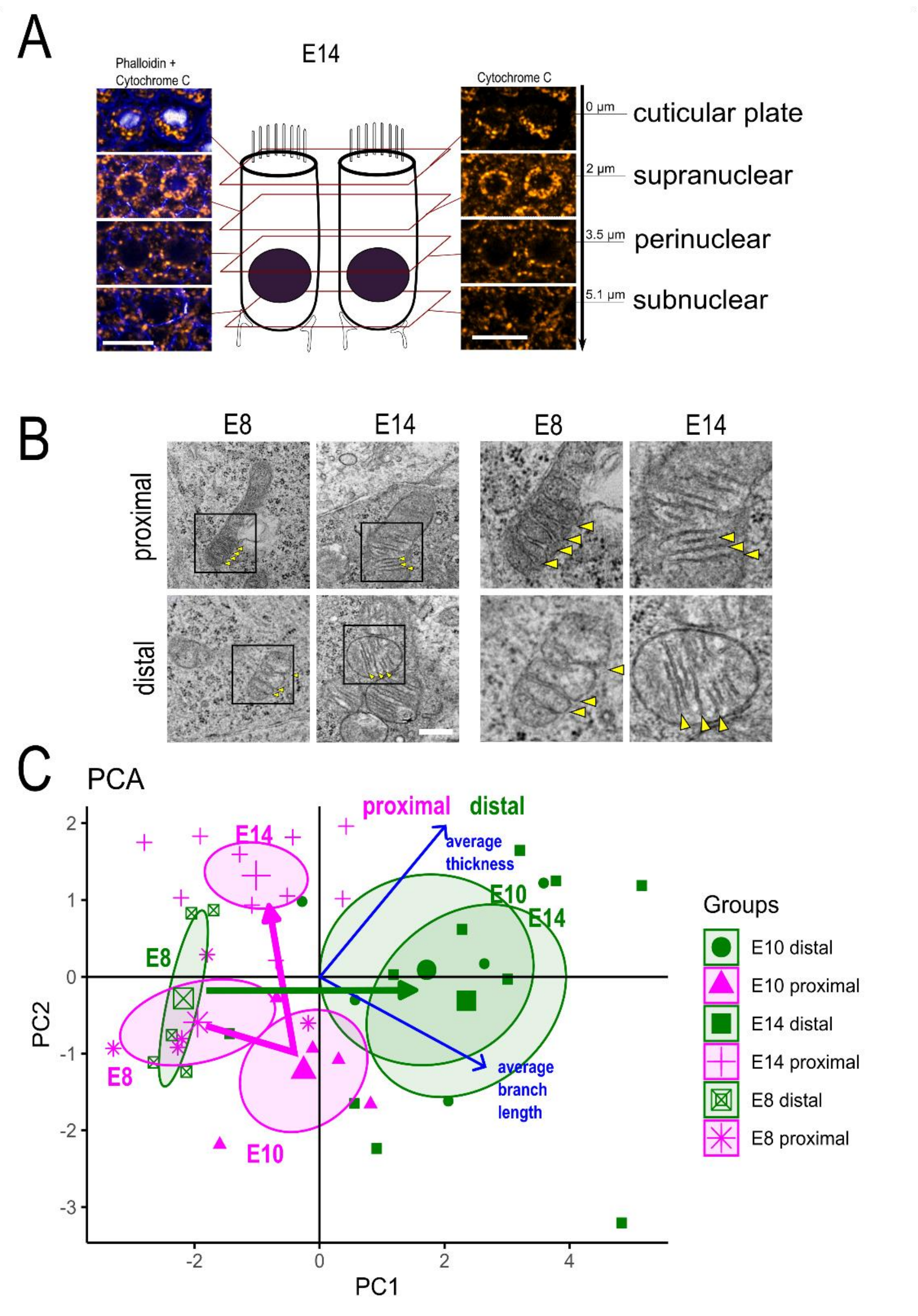

Fig. S1. Mitochondrial morphology in cochlear HCs.
(A) Immunohistochemistry of the mitochondrial antigen cytochrome c (orange), shown in combination with phalloidin (blue) at E14. Optical sections are taken from the different populations of mitochondria along the proximal-distal axis of the HC. Scale = 10 µm.
(B) Left: TEM images of mitochondria within the supranuclear region of HCs at E8 and E14. Yellow arrowheads indicate mitochondrial cristae. Right: illustration of the outlines of mitochondria, and the positions of cristae. Scale = 500 nm.

(C) Principal component analysis (PCA) biplot of local mitochondrial network characteristics in HCs, including directionality of average thickness and branch length measurements (blue arrows). Each datapoint represents one HC sampled from distal (green) or proximal (magenta) regions of the tissues. Larger points represent means, and ovals represent 95% confidence intervals. Magenta and green arrows indicate the morphological trajectories of mitochondrial networks during HC development in proximal and distal regions, respectively.

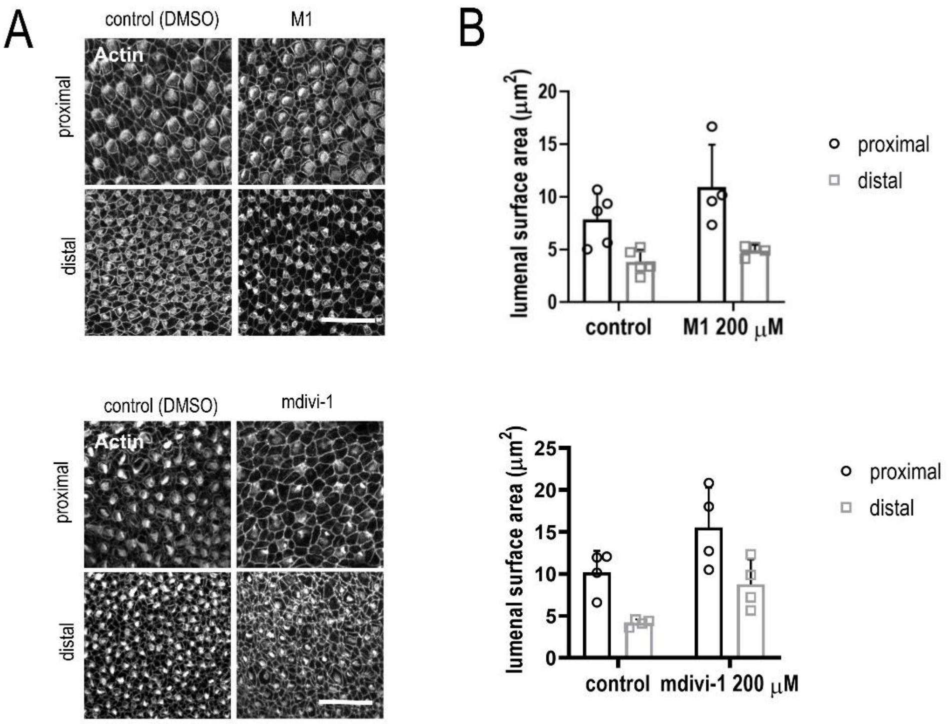

Fig. S2. Individual effects of mdivi-1 and M1 treatment on the lumenal surface area of HCs. Cochleae were dissected from embryonic chickens at E9. The sensory epithelium was isolated and cultured ex vivo for 4 days with small molecule modulators of mitochondrial dynamics. HC apical surface area was delineated using phalloidin staining. **A**) treatment with 200 µM M1 or 200 µM mdivi-1 produced no effect on the lumenal surface area of HCs. Scale = 20 µm. **B**) Quantification of modulator treatment shown in **A**. data are presented as means ± s.d.

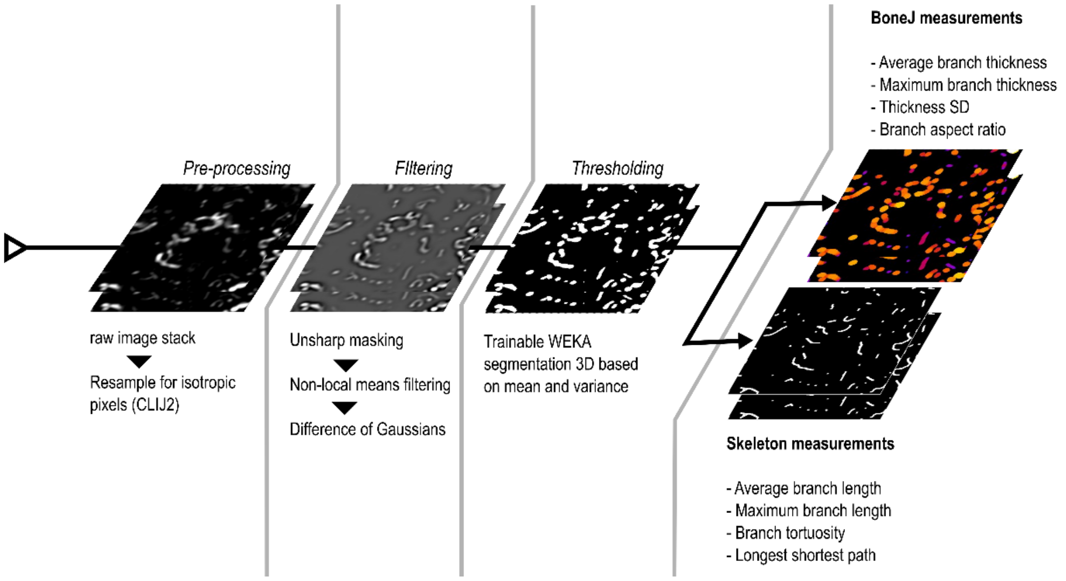

Fig. S3. Image processing and analysis workflow for measurement of mitochondrial morphology.

Development • Supplementary information
