# Supplementary material for: Mitochondrial dynamics regulate cell morphology in the developing cochlea
Source: Development. 2024 Aug 9;151(15):dev202845. doi: 10.1242/dev.202845 (PMC11809207; doi:10.1242/dev.202845)
Supplement: Supplementary file 1 [file develop-151-202845_review_history.pdf]

Development

Mitochondrial dynamics regulate cell morphology in the developing cochlea

James DB O'Sullivan, Stephen Terry, Claire A Scott, Anwen Bullen, Daniel J Jagger and Zoe F Mann

DOI: 10.1242/dev.202845

Editor: Irene Miguel-Aliaga

Review timeline

Original submission:	4 March 2024
Editorial decision:	2 April 2024
First revision received:	7 June 2024
Editorial decision:	1 July 2024
Second revision received:	2 July 2024
Accepted:	8 July 2024

Original submission

First decision letter

MS ID#: DEVELOP/2024/202845

MS TITLE: Mitochondrial dynamics regulate cell size in the developing cochlea

AUTHORS: James DB O'Sullivan, Stephen Terry, Claire A Scott, Anwen Bullen, Daniel J Jagger, and Zoe F Mann

I have now received all the referees' reports on the above manuscript, and have reached a decision. The referees' comments are appended below, or you can access them online: please go to BenchPressand click on the 'Manuscripts with Decisions' queue in the Author Area.

As you will see, the referees express considerable interest in your work, but have some significant criticisms and recommend a substantial revision of your manuscript before we can consider publication. If you are able to revise the manuscript along the lines suggested, which may involve further experiments, I will be happy receive a revised version of the manuscript. Your revised paper will be re-reviewed by one or more of the original referees, and acceptance of your manuscript will depend on your addressing satisfactorily the reviewers' major concerns. Please also note that Development will normally permit only one round of major revision. If it would be helpful, you are welcome to contact us to discuss your revision in greater detail. Please send us a point-by-point response indicating your plans for addressing the referees' comments, and we will look over this and provide further guidance.

Please attend to all of the reviewers' comments and ensure that you clearly highlight all changes made in the revised manuscript. Please avoid using 'Tracked changes' in Word files as these are lost in PDF conversion. I should be grateful if you would also provide a point-by-point response detailing how you have dealt with the points raised by the reviewers in the 'Response to Reviewers' box. If you do not agree with any of their criticisms or suggestions please explain clearly why this is so.

Reviewer 1

© 2024. Published by The Company of Biologists under the terms of the Creative Commons Attribution License (https://creativecommons.org/licenses/by/4.0/).

Advance summary and potential significance to field

Hair cells in the auditory organs of birds and mammals vary in size and shape according to their position along the frequency (tonotopic) axis of the hearing organ. The mechanisms that regulate such precise variations in cell morphology have remained obscure until recently, when the authors of this manuscript demonstrated differences in glucose and mitochondrial metabolism along the tonotopic axis of the bird hearing organ . Here, the authors follow up these results by showing that mitochondrial morphology - a function of the rates of mitochondrial fission and fusion - varies along the tonotopic axis, and that modifying the rates of these processes can change the morphology of hair cells. The results suggest that mitochondrial dynamics are one mechanism by which the size and shape of hair cells are regulated.

Comments for the author

This is a nice follow up to the group's recent 2023 eLife paper on how glucose and mitochondrial metabolism vary across the tonotopic axis of the basilar papilla. The authors have done a very nice job carefully quantifying different parameters of hair cells and their mitochondria across the tonotopic axis and their findings that pharmacologically altering mitochondrial dynamics are convincing. I have a number of suggestions for revisions to make the paper more accessible to readers of Development, who may not be specialists in hair cells in birds.

1. In Figure 1B, it might also be helpful to show a view of the hair cells from above because (a) most of the measurements in the paper are made by imaging whole mounts from above, not in sections and (b) it would be easy to indicate what the authors mean by "lumenal surface area". I know what the authors mean, but non-specialists may be confused! Maybe "apical surface area" might be easier to understand?

2. Because the authors are measuring different parameters of hair cells, they should be clear what exactly they mean by "size", "shape" and "volume" in the text.

3. In addition to differences in hair cell shape along the proximo-distal axis, there are also differences in the neural-abneural axis, where one sees a transition from "tall" to "short" hair cells. The authors don't really mention this in the text, but in the X-axis of figure 1G, the authors show they are comparing volume of proximal short hair cells to distal tall hair cells. Is this a fair comparison? Unless the numbers of SHCs and THCs vary drastically along the P-D axis, shouldn't they compare proximal THC with distal THC and proximal SHC with distal SHC?

4. Panels S1A and B are very small and the text is tiny. Could they be made larger, and also can the authors point out the differences in mitochondrial structure/crista density and thickness, as I am struggling to see them at the moment.

5. For each experiment where the authors do a PCA analysis of mitochondrial characteristics, it would be helpful to state in the main text exactly which parameters were incorporated into the PCA. They sometimes mention this in the figure legends, but being completely clear in the text would be good.

6. Did the authors do TEM or reconstructions of the inhibitor-treated cultures to verify that they are really affecting mitochondrial dynamics? I am not familiar with these inhibitors, so I don't know how well-established their effects are in the literature.

7. (Minor point) In addition to hair cell size, there are also tonotopic variations in stereovilli length. I know this is outside the current scope of the paper, but did the authors see any changes in bundle length when they modulated mitochondrial dynamics? It might be nice to discuss whether cell shape/size can be uncoupled from bundle length.

<u>Reviewer 2</u>

Advance summary and potential significance to field

© 2024. Published by The Company of Biologists under the terms of the Creative Commons Attribution License (https://creativecommons.org/licenses/by/4.0/).

This manuscript by O'Sullivan et al tests the connection between mitochondrial dynamics and cell size in the chick cochlea, where cell sizes associates with the sound frequency these cells respond. The results suggest that mitochondrial dynamics regulate hair cell size. The model is an interesting example of a system where cell size appears to be critical for the proper cell function. This work will be of interest for the broader community as it highlights how cell size specification may be achieved in the tissue leading to functional organization of cells and tissues during development.

Comments for the author

The authors suggest that cell size alterations could occur by altering mitochondrial dynamics. While the cell culture experiments which chemical inhibitors suggest that mitochondrial dynamics regulates cell size, there is a critical issue in the interpretation of the mitochondrial morphology data that needs to be fixed before I can recommend publication of this work.

I am not convinced by the conclusions drawn from Fig 2 PCA plots. It is claimed that the proximal samples of E8-E14 differ significantly base on the 95% CI shown by the ellipses. The scatter of the data is quite high, but my main issue is that if the variables in 2D are are the most important ones contributing to the PC1 and PC2 dimensions, you are not really looking at the mitochondrial morphology but simply increase in mitochondrial volume (which scales proportionally to cell size). Thus it could be that clustering is simply due to increase in cell size, which results in increase in mitochondrial content and has nothing to do with the mitochondrial dynamics, which is hypothesized to be the reason for the increased cell size. I am not asking for causality here as that could work both ways. Instead, to strengthen the idea that the mitochondrial network is important, it would be better to leave out the mitochondrial volume or normalize it to the cell volume to see how the PCA clustering behaves.

Another issue I have is with Fig 3D, the PCA analysis needs some more description to the figure legend or maybe a color coded legend to make it clear as there are many groups. My main question is why was MFN1 omitted from this PCA analysis? If the hypothesis that the mitochondrial characteristics in both explants and cells differ along the same axis, one would expect MFN1 sgRNA samples to fall on this line as well. This would substantially strengthen the conclusion. This PCA analysis also suffers from the same problem that the dominant factor responsible for the directional alignment of the samples could be again the cell size itself.

The methods section is insufficient in depth for replicating this work. I would like to see minimally the parameters used for the PCA clustering as the description of this analysis is extremely brief. The cell volume measurements are also critical and should be described in more detail. The sources of the chemicals should be also listed.

Minor:

Typos: Page 5 says "Principle" Component Analysis (PCA) instead of "Principal"

Page 13 "DF1 cells we purchased directly from ATTC" should be ATCC.

Page10: "However, co-treatment with mdivi-1 and M1 increased the distal HC LSA to levels comparable to those of proximal HCs (Fig 3B, C)." The reference to the figure 3 is wrong.

Reviewer 3

Advance summary and potential significance to field

This manuscript addresses fundamental questions about how the tonotopic organization of auditory hair cells is established in the chick basilar papilla.
Hair cells exhibit several distinct characteristics depending on their position along the tonotopic axis. This study tested the hypothesis that the differences in hair cell size between proximal and distal regions of the BP are controlled by the dynamics of mitochondrial fusion and fission. While this study sheds light on novel aspects of hair cell size control, there are several concerns that need be addressed to strengthen the authors' arguments.

© 2024. Published by The Company of Biologists under the terms of the Creative Commons Attribution License (https://creativecommons.org/licenses/by/4.0/).

Comments for the author

This manuscript addresses fundamental questions about how the tonotopic organization of auditory hair cells is established in the chick basilar papilla. Hair cells exhibit several distinct characteristics depending on their position along the tonotopic axis. This study tested the hypothesis that the differences in hair cell size between proximal and distal regions of the BP are controlled by the dynamics of mitochondrial fusion and fission. While this study sheds light on novel aspects of hair cell size control, there are several concerns that need be addressed to strengthen the authors' arguments.

Major points:

Fig 2. The results show that the mitochondrial activity, as estimated by TMRM fluorescence intensity, does not correlate with mitochondrial volume in developing HCs. This uncoupling is unexpected and different from what has been reported previously. I have two concerns:

First, the sample size of HCs analyzed for fluorescence intensity seems insufficient (3 HCs from 2 BP samples). To determine whether the dissociation between mitochondrial volume and activity is a feature of HC development, it is recommended to increase the number of hair cells (n) in additional biological samples.

Secondly, and more importantly, Fig 2E-F shows that there is a significant difference in TMRM fluorescence intensity between proximal and distal BP at E8 and E10. In contrast, the authors' previous work published in eLife (2023) showed no significant differences in TMRM fluorescence intensity between proximal and distal regions at any developmental stage. This discrepancy must be clearly explained. If there are differences in image processing methods from previous studies, similarly processed images and results consistent with the previous study should be presented.

Fig S2. The data presented in Fig S2 raise several concerns.

1. There appears to be a discrepancy between the brightness of the images in panel A and the fluorescence intensity shown in the graph in panel B. Similarly, between panels 2C and D. I wonder if the images in panels A and C have been accidentally swapped.
2. Differential fluorescence intensity of MFN1 and pDNM1L was used to investigate their expression levels in developing HCs. Please provide references that validate these antibodies and confirm their efficacy in representing the localization and amount of the proteins.
3. Of particular concern is the significant variability in fluorescence intensity even within the same region, with differences of over 100-fold (e.g. MFN1 intensity dots in the E8 distal region), making comparisons of expression levels unreliable.
4. In addition, the appearance of the fluorescence varies from image to image, raising the question of whether the fluorescence actually represents mitochondria. Including mitochondrial markers will help to determine whether the observed immunoreactivities are representative of mitochondria.

Fig 4D. The luminal surface area (LSA) of the proximal control regions also varies significantly between experiments. For example, the LSA of the proximal controls ranged from approximately 7um^2 (mdivi-1+M1 experiment) to 15um^2 (MYLS22 experiment). Due to this large variability, the claim that mdivi-1+M1 treatment increases the LSA is not well supported, as the increased LSA is below 15 um^2, which is well within the control range. These experiments should be performed on the same set (control, mdivi-1+M1, and MYLS22 together) so that the control data can serve as a control for both experiments.

P11. *"Overall, our findings suggest that differences mitochondrial fusion in the supranuclear region of HCs are a driver, rather than a consequence, of the larger high- frequency HC size."* To support this claim, it is necessary to analyze changes in mitochondrial fusion in drug-treated explants. The authors interpret larger mitochondrial volume as an indication of tendency towards fusion. Therefore, changes in LSA induced by mdivi-1+M1 and MYLS22 treatment should correlate with changes in mitochondrial volume.

© 2024. Published by The Company of Biologists under the terms of the Creative Commons Attribution License (https://creativecommons.org/licenses/by/4.0/).

Minor points:

Several typographical mistakes and incorrect figure labels were observed. Please make sure all of these errors are corrected. Here are some examples.

- P.5. “shorter in proximal compared to distal HCs (Fig 2C).” ➔ Fig. 2D
- P2. “significantly larger that the low-frequency distal HCs” ➔ than
- P10. “comparable to those of proximal HCs (Fig 3B, C).” ➔ Fig 4C, D

P3. “biasing towards fusion or fission within a critical window of development alters HC size and tonotopic identity.” Hair cell size is only one aspect of the tonotopic properties, so it is inappropriate to use ‘tonotopic identity’ without analyzing changes in additional tonotopic properties.

Fig 1G. Add an explanation of what SHC and THC stand for to the figure legend.

Fig S1B. Are the mitochondria images from the supranuclear region? Please indicate the sub-population from which the TEM images were taken.

P11. *“These morphological differences manifest as distinct surface-volume (SV) ratios at different tonotopic positions which determine the physiological properties of a HC (Fettiplace and Nam, 2019).”* Please include references that demonstrate with experimental data that SV ratios determine the tonotopic physiological properties.

Fig 4A. Change Drp1 to DNM1L to match with the terminology in the text.

First revision

Author response to reviewers' comments

Dear editor,

We would like to thank the reviewers for their insightful feedback and positive review of our article, highlighting it as an interesting investigation of a poorly understood aspect of tonotopic development presenting novel findings that will be of interest to a broad readership. Based on their comments, we have prepared a revised version of the manuscript in which all concerns raised by the reviewers have been fully addressed. As recommended, we have conducted additional experiments to increase the statistical power of our existing analyses, and further strengthen the methodology of our *in vitro* explant culture experiments. Namely, we show that MYLS22, M1 and mdivi-1 all modulate mitochondrial volume as well as cell morphology. Finally, all typographical corrections have been made and further detail on experimental and methodological procedures have been included as requested. We included a point-by-point response to reviewer’s comments below. All modifications of the text are highlighted in red in the manuscript file.

Reviewer 1 Advance Summary and Potential Significance to Field:
Here, the authors follow up recent work by showing that mitochondrial morphology - a function of the rates of mitochondrial fission and fusion - varies along the tonotopic axis, and that modifying the rates of these processes can change the morphology of hair cells. The results suggest that mitochondrial dynamics are one mechanism by which the size and shape of hair cells are regulated.

Reviewer 1 Comments for the Authors:
This is a nice follow up to the group's recent 2023 eLife paper on how glucose and mitochondrial metabolism vary across the tonotopic axis of the basilar papilla. The authors have done a very nice job carefully quantifying different parameters of hair cells and their mitochondria across the tonotopic axis, and their findings that pharmacologically altering

© 2024. Published by The Company of Biologists under the terms of the Creative Commons Attribution License (https://creativecommons.org/licenses/by/4.0/).

mitochondrial dynamics are convincing.

Suggestions for revisions to make the paper more accessible to readers of Development, who may not be specialists in hair cells in birds:

1. In Figure 1B, it might also be helpful to show a view of the hair cells from above because (a) most of the measurements in the paper are made by imaging whole mounts from above, not in sections and (b) it would be easy to indicate what the authors mean by "lumenal surface area". I know what the authors mean, but non-specialists may be confused! Maybe "apical surface area" might be easier to understand?

As recommended, we have included a surface view of hair cells in fig 1B to depict the morphology of the bird cochlea more clearly. We have also clarified what is meant by the 'lumenal surface area' in the legend for Figure 1 (lines 473-474) and the Introduction (line 55), highlighting its location at the apical pole of the HC.

2. Because the authors are measuring different parameters of hair cells, they should be clear what exactly they mean by "size", "shape" and "volume" in the text.

In response to this recommendation, we have simplified the terminology used to describe cell morphology throughout the manuscript. We hope this addresses the reviewer's concern and provides increased accuracy and clarity for the reader:

- Any references to cell "size" have been removed, as we feel that the term is too vague. In every case this has been replaced with reference to the HC 'lumenal surface area (LSA)', which is a more precise term defined in line 55 and in the Figure 1 legend (lines 473-474).
- Changed terminology from "shape" to "morphology".
- Use "HC volume" only in relation to the calcein measurements (Figure 1G).

3. In addition to differences in hair cell shape along the proximo-distal axis, there are also differences in the neural-abneural axis, where one sees a transition from "tall" to "short" hair cells. The authors don't really mention this in the text, but in the X-axis of figure 1G, the authors show they are comparing volume of proximal short hair cells to distal tall hair cells. Is this a fair comparison? Unless the numbers of SHCs and THCs vary drastically along the P-D axis, shouldn't they compare proximal THC with distal THC and proximal SHC with distal SHC?

We thank the reviewer for raising this important point and apologise for this error on our part. For all imaging and analysis, we sampled from regions centrally along the mid-line of the tissue (red boxes, Figure 1). This was done to maintain consistency between our measurements. Using this method of sampling, both SHCs and THCs populations are captured within a single field of view in both proximal and distal regions. We are therefore confident our analysis accurately captures and interrogates differences in cell morphology across both populations along the epithelium.

Further to this, and addressing the concern raised by the reviewer, we have amended panel G in Figure 1 and removed reference to THCs and SHCs.

4. Panels S1A and B are very small and the text is tiny. Could they be made larger, and also can the authors point out the differences in mitochondrial structure/crista density and thickness, as I am struggling to see them at the moment.

We have increased the size of the panels and text to aid legibility. Furthermore, we have added higher magnification insets to each panel to better highlight the structure and density of the cristae (Figure S1B).

5. For each experiment where the authors do a PCA analysis of mitochondrial characteristics, it would be helpful to state in the main text exactly which parameters were incorporated into the PCA. They sometimes mention this in the figure legends, but being

© 2024. Published by The Company of Biologists under the terms of the Creative Commons Attribution License (https://creativecommons.org/licenses/by/4.0/).

completely clear in the text would be good.

Addressing a concern raised by all 3 reviewers, we have included a complete list of the parameters used for the PCA in the results section of the main article **(lines 127-133)** and in the methods section **(lines 305-310).** The revised description of PCA parameters in the results section now reads:

"Raw fluorescence intensity data were processed and segmented to generate 3D mitochondrial networks (Fig S3) from which average branch thickness, standard deviation of branch thickness, maximum branch thickness, average branch length, maximum branch length, longest shortest path, Euclidean distance (a measure of branch tortuosity), and branch aspect ratio were calculated. Principal Component Analysis (PCA) (Wiemerslage and Lee, 2016) of these seven measurements revealed that HC mitochondria developed along distinct morphological trajectories in proximal and distal regions (Fig 2A - D, Fig S1C)."

6. Did the authors do TEM or reconstructions of the inhibitor-treated cultures to verify that they are really affecting mitochondrial dynamics? I am not familiar with these inhibitors, so I don't know how well- established their effects are in the literature.

The effects of these inhibitors are well-established in the literature (Baek et al., 2017; Manczak et al., 2019). To validate all inhibitors treatments and to confirm that they elicited had the expected effects, we have replicated all *in vitro* experiment in which tissue explants were treated with MYLS22 and M1+mdivi-1. To address the reviewer's concerns, in these experiments, in addition to measuring changes in the HC LSA, we also measured changes in mitochondrial area using immunofluorescence of complex 1. We found that an increase in mitochondrial area in the supranuclear region strongly correlated with increase HC lumenal surface area **(Figure 4 E, F).**

7. (Minor point) In addition to hair cell size, there are also tonotopic variations in stereovilli length. I know this is outside the current scope of the paper, but did the authors see any changes in bundle length when they modulated mitochondrial dynamics? It might be nice to discuss whether cell shape/size can be uncoupled from bundle length.

We agree with the reviewer that these measurements would represent an interesting area of investigation for future work. Due to the focus on size and morphology of the HC body, we felt that investigating stereocilial morphology was beyond the scope of the current article. Additionally, as stereocilial morphology can change in long-term explant culture it may be difficult to separate out effects due to culture and those due to perturbed mitochondrial dynamics. Future studies investigating stereocilial morphology would likely require a genetic approach *in ovo*. Therefore, we have now added a short section to the results/discussion section highlighting that this as an important area for future investigations (lines 215-219).

Reviewer 2 Advance Summary and Potential Significance to Field:
These results suggest that mitochondrial dynamics regulate hair cell size. The model is an interesting example of a system where cell size appears to be critical for the proper cell function. This work will be of interest for the broader community as it highlights how cell size specification may be achieved in the tissue leading to functional organization of cells and tissues during development.

<u>Reviewer 2 Comments for the Authors:</u>
The authors suggest that cell size alterations could occur by altering mitochondrial dynamics. While the cell culture experiments which chemical inhibitors suggest that mitochondrial dynamics regulates cell size, there is a critical issue in the interpretation of the mitochondrial morphology data that needs to be fixed before I can recommend publication of this work.

The scatter of the data is quite high, but my main issue is that if the variables in 2D are the most important ones contributing to the PC1 and PC2 dimensions, you are not really looking at the mitochondrial morphology but simply increase in mitochondrial volume (which scales proportionally to cell size). Thus it could be that clustering is simply due to increase in cell size, which results in increase in mitochondrial content and has nothing to do with the mitochondrial dynamics, which is hypothesized to be the reason for the increased cell size. I am not asking for

© 2024. Published by The Company of Biologists under the terms of the Creative Commons Attribution License (https://creativecommons.org/licenses/by/4.0/).

causality here as that could work both ways. Instead, to strengthen the idea that the mitochondrial network is important, it would be better to leave out the mitochondrial volume or normalize it to the cell volume to see how the PCA clustering behaves.

We would like to emphasise that mitochondrial volume was **not** one of the parameters included in the PCA for the exact reason highlighted by the reviewer. A detailed list of all input parameters is now clearly defined and stated in the methods section **(lines 305-310)** and in the results **(lines 127-133)**. To avoid any additional confusion for readers the legend for figure 2D that erroneously implies volume was included in the PCA has been modified accordingly. We can assure the reviewer that mitochondrial volume was determined independently from the PCA. This is an important and valid concern, and we apologise for not having made the clustering criteria clearer in our original submission.

Another issue I have is with Fig 3D, the PCA analysis needs some more description to the figure legend or maybe a color-coded legend to make it clear as there are many groups. My main question is why was MFN1 omitted from this PCA analysis? If the hypothesis that the mitochondrial characteristics in both explants and cells differ along the same axis, one would expect MFN1 sgRNA samples to fall on this line as well. This would substantially strengthen the conclusion. This PCA analysis also suffers from the same problem that the dominant factor responsible for the directional alignment of the samples could be again the cell size itself.

MFN1 was excluded from this analysis, due to the lack of the treatment's effect on cell area or mitochondrial volume as shown in the results. This lack of difference may be due to redundancy of activity with MFN2 (Chen et al., 2003a), or perhaps cell-specific variation in MFN activity. In practice, other researchers have also found that abrogation of MFN1 does not always result in any alteration of mitochondrial morphology (Hsiao et al., 2021; Lee et al., 2012).

The methods section is insufficient in depth for replicating this work. I would like to see minimally the parameters used for the PCA clustering as the description of this analysis is extremely brief. The cell volume measurements are also critical and should be described in more detail. The sources of the chemicals should be also listed.

All parameters for PCA clustering have been included and clearly stated in the methods section, **lines 305-310** and for further clarity, we have also now listed these in the results section, **lines 127-133**. The description of cell volume measurements in the methods has been supplemented with additional detail (lines 279-286), and a citation of other primary literature using a similar protocol for reference. The sources for all chemicals are now listed clearly in the appropriate methods sections.

Minor:

Typos: Page 5 says "Principle" Component Analysis (PCA) instead of "Principal"

Thank you for pointing out this typographic error. It has now been corrected.

Page 13 "DF1 cells we purchased directly from ATTC" should be ATCC.

This spelling has now been corrected.

Page10: "However, co-treatment with mdivi-1 and M1 increased the distal HC LSA to levels comparable to those of proximal HCs (Fig 3B, C)." The reference to the figure 3 is wrong.

This reference has now been amended.

Reviewer 3 Advance Summary and Potential Significance to Field:
This manuscript addresses fundamental questions about how the tonotopic organization of auditory hair cells is established in the chick basilar papilla. Hair cells exhibit several distinct characteristics depending on their position along the tonotopic axis. This study tested the hypothesis that the differences in hair cell size between proximal and distal regions of the BP are controlled by the dynamics of mitochondrial fusion and fission.

© 2024. Published by The Company of Biologists under the terms of the Creative Commons Attribution License (https://creativecommons.org/licenses/by/4.0/).

While this study sheds light on novel aspects of hair cell size control, there are several concerns that need be addressed to strengthen the authors' arguments.

Reviewer 3 Comments for the Authors:

Major points:

Fig 2. The results show that the mitochondrial activity, as estimated by TMRM fluorescence intensity, does not correlate with mitochondrial volume in developing HCs. This uncoupling is unexpected and different from what has been reported previously. I have two concerns:

First, the sample size of HCs analyzed for fluorescence intensity seems insufficient (3HCs from 2 BP samples). To determine whether the dissociation between mitochondrial volume and activity are a feature of HC development, it is recommended to increase the number of hair cells (n) in additional biological samples.

Additional biological replicates have been conducted and added to the dataset, which now includes measurements from 6 HCs taken from 4 independent biological replicates. We are confident that that the comparison of TMRM fluorescence between proximal and distal HCs has been conducted in a robust and reproducible manner (see revised **Figure 2F and legend**).

Secondly, and more importantly, Fig 2E-F shows that there is a significant difference in TMRM fluorescence intensity between proximal and distal BP at E8 and E10. In contrast, the authors' previous work published in eLife (2023) showed no significant differences in TMRM fluorescence intensity between proximal and distal regions at any developmental stage. This discrepancy must be clearly explained. If there are differences in image processing methods from previous studies, similarly processed images and results consistent with the previous study should be presented.

The reviewer has raised a valid concern about an apparent discrepancy in TMRM measurements between studies. We would like to emphasise that between the two studies, the TMRM images were acquired using different imaging acquisition and analysis pipelines, ensuing important differences in how the two datasets should be interpreted. In our 2023 study in ELife, we sought to correlate TMRM and 2-NBDG fluorescence with NAD(P)H FLIM measurements made in proximal and distal regions of the BP. In these experiments it was not possible to acquire the two signals simultaneously nor obtain single cell resolution using NAD(P)H FLIM, and therefore the acquisition conditions and analysis of all three fluorescent signals was kept as consistent as possible across different microscopes. For quantification of NAD(P)H FLIM, 2-NBDG and TMRM all measurements were made from 100 μm x 100 μm ROIs in the proximal and distal regions of the tissue. The measurements from O'Sullivan et al 2023 were therefore not cell specific at E7 and E9 and instead represent the total fluorescence measured from a population of cells containing both HCs and SCs. In the present manuscript under consideration, measurements of mitochondrial morphology and TMRM fluorescence were made specifically from mitochondria in single HCs. This was possible due to the super-resolution capabilities of a newly acquired Zeiss 980 Airyscan 2 microscope. We therefore maintain that the HC-specific differences in TMRM fluorescence presented in fig 2 E-F are not in conflict with the lower resolution, cell population measurements presented in our previous study.

Fig S2.
The data presented in Fig S2 raise several concerns.

1. There appears to be a discrepancy between the brightness of the images in panel A and the fluorescence intensity shown in the graph in panel B. Similarly, between panels 2C and D. I wonder if the images in panels A and C have been accidentally swapped.

Author note to editor and reviewer 3: For our response to all comments relating to figure S2 (points 1. To 4.), please see point 4.

2. Differential fluorescence intensity of MFN1 and pDNM1L was used to investigate their expression levels in developing HCs. Please provide references that validate these antibodies and confirm their efficacy in representing the localization and amount of the proteins.

© 2024. Published by The Company of Biologists under the terms of the Creative Commons Attribution License (https://creativecommons.org/licenses/by/4.0/).

Please see point 4 below.

3. Of particular concern is the significant variability in fluorescence intensity even within the same region, with differences of over 100-fold (e.g. MFN1 intensity dots in the E8 distal region), making comparisons of expression levels unreliable.

Please see point 4 below.

4. In addition, the appearance of the fluorescence varies from image to image, raising the question of whether the fluorescence actually represents mitochondria. Including mitochondrial markers will help to determine whether the observed immunoreactivities are representative of mitochondria.

We thank the reviewer for raising this important point. MFN1 is thought to be purely mitochondrially localised, whereas DNM1L has a large cytoplasmic staining component (Chen et al., 2003b). After deliberating the issue raised by the reviewer, we have performed additional experiments and determined that it is not possible to robustly quantify in a HC-specific manner the levels of mitochondrially-associated pDNM1L immunoreactivity. It was not possible to optically exclude the cytoplasmic pDNM1L signal, which is presumably not actively involved in the fission process, with sufficient accuracy. This was owing to the high abundance of mitochondria in HCs. For these reasons, in addition to the peripheral importance of this supplementary figure in comparison to our other descriptive results and functional perturbation experiments, these immunofluorescence data are no longer included in the manuscript.

Fig 4D. The luminal surface area (LSA) of the proximal control regions also varies significantly between experiments. For example, the LSA of the proximal controls ranged from approximately 7um2 (mdivi-1+M1 experiment) to 15um2 (MYLS22 experiment). Due to this large variability, the claim that mdivi-1+M1 treatment increases the LSA is not well supported, as the increased LSA is below 15 um2, which is well within the control range. These experiments should be performed on the same set (control, mdivi-1+M1, and MYLS22 together) so that the control data can serve as a control for both experiments.

We thank the reviewer for their close examination of this figure. In explant culture, some degree of variation in final lumenal surface area (LSA) of proximal HCs is expected, and we see that this variation of HC size is in accordance with previous explant culture experiments carried out by the investigators (Mann et al., 2014; Thiede et al., 2014), and indeed is in line with our previous culture results in O'Sullivan et al. (2023). We believe that this phenomenon is because proximal HCs are the cell population experiencing the most dramatic morphological changes and their growth may therefore be more sensitive to subtle differences in culture conditions. We acknowledge that the range in proximal values specified by the reviewer (7 μm^2 - 15 μm^2) accurately reflects how proximal HC surface area can vary over the culture window. However, we would like to note two key factors which support our interpretation of this data:

1) That the distal cell size remains extremely conserved across all treatment groups apart form M1+mdivi-1. Control cultures in the first submission of the manuscript for M1, mdivi-1, and MYLS22 all have distal HC sizes clustered just below 5 μm^2. The only group in which this diverges is the M1+mdivi-1 group, in which there is a significant increase (original submission Fig 4 D). The divergence of the distal HC surface area to well above this conserved 5 μm^2 limit is the key result indicating that fusion can increase HC LSA, and its interpretation is not impacted by variation of proximal HC LSA measurements from a different region of the tissue.
2) To the above, we would also add that upon treatment with MYLS22, there is a significant reduction in proximal HC LSA to below 7 um2, the average size of proximal HCs in the control experiments for M1, mdivi1, and M1+Mdivi1 groups.

As suggested by the reviewer, we have carried out a replicate experiment using a common control group for both experimental treatments, so all cultures were carried out on the same day,

© 2024. Published by The Company of Biologists under the terms of the Creative Commons Attribution License (https://creativecommons.org/licenses/by/4.0/).

minimising any variability in tissue handling and culture conditions. The data content of Figure 4 has been replaced by data from this updated and improved experiment, which supports the original conclusions of the first version of the manuscript.

P11. *"Overall, our findings suggest that differences mitochondrial fusion in the supranuclear region of HCs are a driver, rather than a consequence, of the larger highfrequency HC size."* To support this claim, it is necessary to analyze changes in mitochondrial fusion in drug-treated explants. The authors interpret larger mitochondrial volume as an indication of tendency towards fusion. Therefore, changes in LSA induced by mdivi-1+M1 and MYLS22 treatment should correlate with changes in mitochondrial volume.

In order to confirm that the inhibitors were having the intended effect, we replicated the *in vitro* experiment in which tissue explants were treated with MYLS22 and M1+mdivi-1, as per the last point raised by the reviewer. However, in addition to measuring changes in the cell surface area, we estimated changes in mitochondrial abundance using immunofluorescence of complex 1. We found that changes in HC mitochondrial area in the supranuclear region strongly correlated with changes in HC LSA (Fig 4 E-F)

Minor points:
Several typographical mistakes and incorrect figure labels were observed.
Please make sure all of these errors are corrected. Here are some examples.

- P.5. "shorter in proximal compared to distal HCs (Fig 2C)."
  Fig. 2D

Corrected

- P2. "significantly larger that the low-frequency distal HCs"
  than

Corrected

- P10. "comparable to those of proximal HCs (Fig 3B, C)."
  Fig 4C, D

Corrected

P3. "biasing towards fusion or fission within a critical window of development alters HC size and tonotopic identity." Hair cell size is only one aspect of the tonotopic properties, so it is inappropriate to use 'tonotopic identity' without analyzing changes in additional tonotopic properties.

We have removed reference to tonotopic identity, as examining other tonotopic properties of the HCs is beyond the scope of this work. A short reference to how the effects of mitochondrial dynamics on tonotopic identity as an interesting area for future investigation has been added at the end of the results/discussion (Lines 215-219).

Fig 1G. Add an explanation of what SHC and THC stand for to the figure legend.

These terms have been removed to aid clarity, in agreement with comments from Reviewer 1.

Fig S1B. Are the mitochondria images from the supranuclear region?

Please indicate the sub-population from which the TEM images were taken.

We have indicated that measurements were taken from the supranuclear region (Legend Fig S1).

P11. *"These morphological differences manifest as distinct surface-volume (SV) ratios at different tonotopic positions which determine the physiological properties of a HC (Fettiplace and Nam, 2019)."*

© 2024. Published by The Company of Biologists under the terms of the Creative Commons Attribution License (https://creativecommons.org/licenses/by/4.0/).

Please include references that demonstrate with experimental data that SV ratios determine the tonotopic physiological properties.

There are currently no experimental/electrophysiological data showing a direct functional link between the S-V ratio and the HC physiological tuning properties in birds or reptiles. There are however numerous studies showing that in mammals, HC size correlates with frequency position along the basal-to-apical axis of the cochlea. We have re-phrased this section accordingly and as requested included the supporting references (Fettiplace, 2017; Fettiplace and Nam, 2019; Johnson et al., 2011; Pujol et al., 1992). Please refer to lines 208-219 for revised text.

Fig 4A. Change Drp1 to DNM1L to match with the terminology in the text.

This erroneous rodent protein name has now been corrected to the appropriate avian protein name in Fig 4.

References:

Baek, S.H., Park, S.J., Jeong, J.I., Kim, S.H., Han, J., Kyung, J.W., Baik, S.-H., Choi, Y., Choi, B.Y., Park, J.S., Bahn, G., Shin, J.H., Jo, D.S., Lee, J.-Y., Jang, C.-G., Arumugam, T.V., Kim, J., Han, J.-W., Koh, J.-Y.,Cho, D.-H., Jo, D.-G., 2017. Inhibition of Drp1 Ameliorates Synaptic Depression, Aß Deposition, and Cognitive Impairment in an Alzheimer's Disease Model. J Neurosci 37, 5099–5110. https://doi.org/10.1523/JNEUROSCI.2385-16.2017

Chen, H., Detmer, S.A., Ewald, A.J., Griffin, E.E., Fraser, S.E., Chan, D.C., 2003a. Mitofusins Mfn1 and Mfn2 coordinately regulate mitochondrial fusion and are essential for embryonic development. J Cell Biol 160, 189-200. https://doi.org/10.1083/jcb.200211046

Chen, H., Detmer, S.A., Ewald, A.J., Griffin, E.E., Fraser, S.E., Chan, D.C., 2003b. Mitofusins Mfn1 and Mfn2 coordinately regulate mitochondrial fusion and are essential for embryonic development. J Cell Biol 160, 189-200. https://doi.org/10.1083/jcb.200211046

Fettiplace, R., 2017. Hair cell transduction, tuning and synaptic transmission in the mammalian cochlea. Compr Physiol 7, 1197–1227. https://doi.org/10.1002/cphy.c160049

Fettiplace, R., Nam, J.-H., 2019. Tonotopy in calcium homeostasis and vulnerability of cochlear hair cells. Hearing Research, Annual Reviews 2019 376, 11–21. https://doi.org/10.1016/j.heares.2018.11.002 Hsiao, Y.T., Shimizu, I., Wakasugi, T., Yoshida, Y., Ikegami, R., Hayashi, Y., Suda, M., Katsuumi, G., Nakao, M., Ozawa, T., Izumi, D., Kashimura, T., Ozaki, K., Soga, T., Minamino, T., 2021. Cardiac mitofusin-1 is reduced in non-responding patients with idiopathic dilated cardiomyopathy. Sci Rep 11, 6722. https://doi.org/10.1038/s41598-021-86209-y

Johnson, S.L., Beurg, M., Marcotti, W., Fettiplace, R., 2011. Prestin-driven cochlear amplification Is not limited by the outer hair cell membrane time constant. Neuron 70, 1143–1154. https://doi.org/10.1016/j.neuron.2011.04.024

Lee, S., Sterky, F.H., Mourier, A., Terzioglu, M., Cullheim, S., Olson, L., Larsson, N.-G., 2012. Mitofusin 2 is necessary for striatal axonal projections of midbrain dopamine neurons. Human Molecular Genetics 21, 4827-4835. https://doi.org/10.1093/hmg/dds352

Manczak, M., Kandimalla, R., Yin, X., Reddy, P.H., 2019. Mitochondrial division inhibitor 1 reduces dynamin- related protein 1 and mitochondrial fission activity. Hum Mol Genet 28, 177–199. https://doi.org/10.1093/hmg/ddy335

Mann, Z.F., Thiede, B.R., Chang, W., Shin, J.-B., May-Simera, H.L., Lovett, M., Corwin, J.T., Kelley, M.W., 2014. A gradient of Bmp7 specifies the tonotopic axis in the developing inner ear. Nat Commun 5, 3839. https://doi.org/10.1038/ncomms4839

Pujol, R., Lenoir, M., Ladrech, S., Tribillac, F., Rebillard, G., 1992. Correlation Between the Length of Outer Hair Cells and the Frequency Coding of the Cochlea, in: Cazals, Y., Horner, K., Demany, L. (Eds.), Auditory Physiology and Perception. Pergamon, pp. 45-52. https://doi.org/10.1016/B978-0-08-041847-6.50011-3

Thiede, B.R., Mann, Z.F., Chang, W., Ku, Y.-C., Son, Y.K., Lovett, M., Kelley, M.W., Corwin, J.T., 2014. Retinoic acid signalling regulates the development of tonotopically patterned hair cells in the chicken cochlea. Nat Commun 5, 3840. https://doi.org/10.1038/ncomms4840

Wiemerslage, L., Lee, D., 2016. Quantification of Mitochondrial Morphology in Neurites of Dopaminergic Neurons using Multiple Parameters. J Neurosci Methods 262, 56–65. https://doi.org/10.1016/j.jneumeth.2016.01.008

© 2024. Published by The Company of Biologists under the terms of the Creative Commons Attribution License (https://creativecommons.org/licenses/by/4.0/).

Second decision letter

MS ID#: DEVELOP/2024/202845

MS TITLE: Mitochondrial dynamics regulate cell morphology in the developing cochlea

AUTHORS: James DB O'Sullivan, Stephen Terry, Claire A Scott, Anwen Bullen, Daniel J Jagger, and Zoe F Mann

I have now received all the referees reports on the above manuscript, and have reached a decision. The referees' comments are appended below, or you can access them online: please go to BenchPress and click on the 'Manuscripts with Decisions' queue in the Author Area.

The overall evaluation is positive and we would like to publish a revised manuscript in Development, provided that the referees' comments can be satisfactorily addressed. Please attend to all of the reviewers' comments in your revised manuscript and detail them in your point-by-point response. If you do not agree with any of their criticisms or suggestions explain clearly why this is so. If it would be helpful, you are welcome to contact us to discuss your revision in greater detail. Please send us a point-by-point response indicating your plans for addressing the referees' comments, and we will look over this and provide further guidance.

Reviewer 1

Advance summary and potential significance to field

Hair cells in the auditory organs of birds and mammals vary in size and shape according to their position along the frequency (tonotopic) axis of the hearing organ. The mechanisms that regulate such precise variations in cell morphology have remained obscure until recently, when the authors of this manuscript demonstrated differences in glucose and mitochondrial metabolism along the tonotopic axis of the bird hearing organ . Here, the authors follow up these results by showing that mitochondrial morphology - a function of the rates of mitochondrial fission and fusion - varies along the tonotopic axis, and that modifying the rates of these processes can change the morphology of hair cells. The results suggest that mitochondrial dynamics are one mechanism by which the size and shape of hair cells are regulated.

Comments for the author

The authors have addressed my previous comments and I have no further concerns with this lovely paper.

Reviewer 2

Advance summary and potential significance to field

These results suggest that mitochondrial dynamics regulate size and shape of hair cells. The model is an interesting example of a system where cell size appears to be critical for the proper cell function. This work will be of interest for the broader community as it highlights how cell size and shape specification may be achieved in the tissue leading to functional organization of cells and tissues during development.

Comments for the author

© 2024. Published by The Company of Biologists under the terms of the Creative Commons Attribution License (https://creativecommons.org/licenses/by/4.0/).

The manuscript has now a more detailed description of the measurements used for the input for the PCA analysis and additional detail on cell size measurements as well. However, despite that response letter saying so, I could not find any mention of the sources for the mitochondrial drugs in the methods section. I also noticed that two whole paragraphs of the methods present in the previous version of the manuscript have gone missing (DF-1 cell and CRISPR method paragragphs).

Reviewer 3

Advance summary and potential significance to field

.

Comments for the author

The authors have thoroughly addressed all of my comments, and the revised manuscript is now ready for publication in Development.

Second revision

Author response to reviewers' comments

Dear Editor,
We thank the editor and reviewers for their positive response to our revised manuscript.
Reviewer 1: The authors have addressed my previous comments and I have no further concerns with this lovely paper.
Reviewer 2: The model is an interesting example of a system where cell size appears to be critical for the proper cell function. This work will be of interest for the broader community as it highlights how cell size and shape specification may be achieved in the tissue leading to functional organization of cells and tissues during development.
Reviewer 3: The authors have thoroughly addressed all of my comments, and the revised manuscript is now ready for publication in Development.
We included below a point-by-point response to reviewer's comments below. All modifications of the text are highlighted in red in the manuscript file.
Reviewer 2: The manuscript has now a more detailed description of the measurements used for the input for the PCA analysis and additional detail on cell size measurements as well. However, despite that response letter saying so, I could not find any mention of the sources for the mitochondrial drugs in the methods section. I also noticed that two whole paragraphs of the methods present in the previous version of the manuscript have gone missing (DF-1 cell and CRISPR method paragragphs).
Line 266: We thank the reviewer for drawing our attention to this omission. The sources for each of the mitochondrial drugs used are listed in full.

Lines 229-259: We again thank the reviewer for noticing this omission error in our previous draft and have re-added the paragraph outlining DF-1 cell line culture and CRISPR methodology. We apologise for this omission.

Third decision letter

MS ID#: DEVELOP/2024/202845

MS TITLE: Mitochondrial dynamics regulate cell morphology in the developing cochlea

© 2024. Published by The Company of Biologists under the terms of the Creative Commons Attribution License (https://creativecommons.org/licenses/by/4.0/).

AUTHORS: James DB O'Sullivan, Stephen Terry, Claire A Scott, Anwen Bullen, Daniel J Jagger, and Zoe F Mann

I am happy to tell you that your manuscript has been accepted for publication in Development, pending our standard publication integrity checks.

© 2024. Published by The Company of Biologists under the terms of the Creative Commons Attribution License (https://creativecommons.org/licenses/by/4.0/).
